# Transcriptional silencing in *Saccharomyces cerevisiae*: known unknowns

**DOI:** 10.1186/s13072-024-00553-7

**Published:** 2024-09-14

**Authors:** Namrita Dhillon, Rohinton T. Kamakaka

**Affiliations:** 1 Department of Biomolecular Engineering, University of California, 1156 High Street, Santa Cruz, CA 95064 USA; 2 Department of MCD Biology, University of California, 1156 High Street, Santa Cruz, CA 95064 USA

## Abstract

Transcriptional silencing in *Saccharomyces cerevisiae* is a persistent and highly stable form of gene repression. It involves DNA silencers and repressor proteins that bind nucleosomes. The silenced state is influenced by numerous factors including the concentration of repressors, nature of activators, architecture of regulatory elements, modifying enzymes and the dynamics of chromatin.Silencers function to increase the residence time of repressor Sir proteins at silenced domains while clustering of silenced domains enables increased concentrations of repressors and helps facilitate long-range interactions. The presence of an accessible NDR at the regulatory regions of silenced genes, the cycling of chromatin configurations at regulatory sites, the mobility of Sir proteins, and the non-uniform distribution of the Sir proteins across the silenced domain, all result in silenced chromatin that only stably silences weak promoters and enhancers via changes in transcription burst duration and frequency.These data collectively suggest that silencing is probabilistic and the robustness of silencing is achieved through sub-optimization of many different nodes of action such that a stable expression state is generated and maintained even though individual constituents are in constant flux.

The DNA in a eukaryotic nucleus is wrapped around histones to form nucleosomes. The interplay between non-histone proteins and nucleosomal filaments leads to stable programs of gene expression [1] resulting in a continuum of expression levels [2–4]. Transcriptional activation involves sequence specific transcription factors (TFs) and general transcription factors (GTFs) [5, 6]. The former bind upstream activating sequence (UAS) enhancers to regulate transcription while the GTFs bind sequences in the core promoter to initiate transcription. Transcriptional repression refers to an inactive state dependent upon the continual presence of the repressing signal and specific repressor proteins that often bind specific sequences [7, 8]. Silencing is a position-independent persistent and stable form of repression that requires proteins that bind to DNA sequences called silencers as well as repressor proteins that interact with nucleosomes to create a structure that silences multiple genes with diverse regulatory elements and once established the expression state is maintained and propagated with high fidelity [9].

The aim of this review is to highlight recent advances and key outstanding questions on gene silencing in *Saccharomyces cerevisiae.* It is not a comprehensive review of the subject and the reader is directed to several excellent comprehensive reviews on this subject [9–13]. There are many parallels between Sir mediated silencing in *S. cerevisiae* and HP1 and Polycomb mediated silencing in other eukaryotes and some of these similarities will be highlighted in the course of this review (also see [14–18]).

## What is Silencing?

Based on recent and prior results:

1) Silencers are critical in establishing and maintaining silencing. Their function is to increase the concentration of the repressor Sir proteins in the vicinity of the silenced chromatin domains.

2) The regulatory elements involved in gene activation are key arbiters that determine the effectiveness of silencing and only weak constitutively active genes are stably and effectively silenced.

3) Silencing is the result of a web of multivalent stereo-specific interactions between Sir proteins, silencer bound proteins and nucleosomes. These interactions create a chromatin environment that reduces nucleosome mobility and hinders the rate of factor access with attendant negative effects on transcription.

4) Silencing is influenced by clustering of silenced domains. The formation of membrane-less compartments increases Sir protein concentration; helps mediate long-range Sir-nucleosome interactions and possibly hinders the ability of cofactors to function.

5) Silencing is an energy dissipating, non-equilibrium, stochastic probabilistic process driven by constituents that are constantly in flux. The process is governed in part by concentration, affinity and residence times of many different individual components. These different parameters together determine the silenced state of a gene.

6) The stability of the silenced state is likely to be governed by diffuse forms of “co-operativity” resulting in elevated avidity of the Sir proteins across the entire domain.

Silencing has been investigated using a myriad of approaches, each of which interrogates distinct and specific aspects of silencing. The individual vignettes integrated together generate a picture of the process. The principles of silenced chromatin gleaned and enunciated in this review, the known unknowns highlighted and the models proposed to explain silenced chromatin are focused on *S. cerevisiae* silencing. The experiments and the models proposed often rely on studies on facultative and constitutive silenced chromatin in other eukaryotes and are also arguably relevant for studies in these organisms.

## Transcription activation

Gene regulation is mediated by proteins that bind specific DNA sequences as well as proteins that interact with and alter the chromatin template and involves tens of molecular reactions. Transcription of a gene is initiated when a site in the enhancer is bound by TFs. Understanding how TFs find and bind DNA is necessary for the understanding of silencing since silencers are bound by proteins that follow the same rules as TF binding to enhancers. Most TFs recognize and bind short DNA sequences and explore the genome through transient interactions until they find and bind a sequence with high affinity for significant periods resulting in “sequence-specific” binding [19–25].

TFs bind DNA sites in the genome with varying strengths, specificities and lengths of time and these interactions then result in functional consequences. Their function is dependent upon their binding domains, their ability to phase separate into condensates, the number of high affinity binding sites for TFs, the density of binding sites, cooperativity of binding and the ability of TFs to interact with multiple components such as other TFs, cofactors, histone modifiers and chromatin remodellers. TF binding can thus be regulated by altering their concentration, biochemical characteristics or binding affinities or by altering their access to binding sites. The residence times of TFs at these elements are also influenced by other factors such as adjacent flanking sequences, chromatin dynamics and silencing [24, 26–34].

While sequence specific interactions are critical for gene regulation, non-specific interactions also play a supporting but important role. The association and dissociation of TFs between clusters of binding sites can trap TFs in a specific region of the nucleus creating membrane-less nuclear compartments with high local concentrations of TFs [26, 35]. The presence of elevated levels of specific proteins in the nucleoplasm can also lead to negative effects by inducing unbinding of TFs via facilitated dissociation akin to squelching [36], a mechanism that could play a role in gene silencing.

**Fig. 1:**
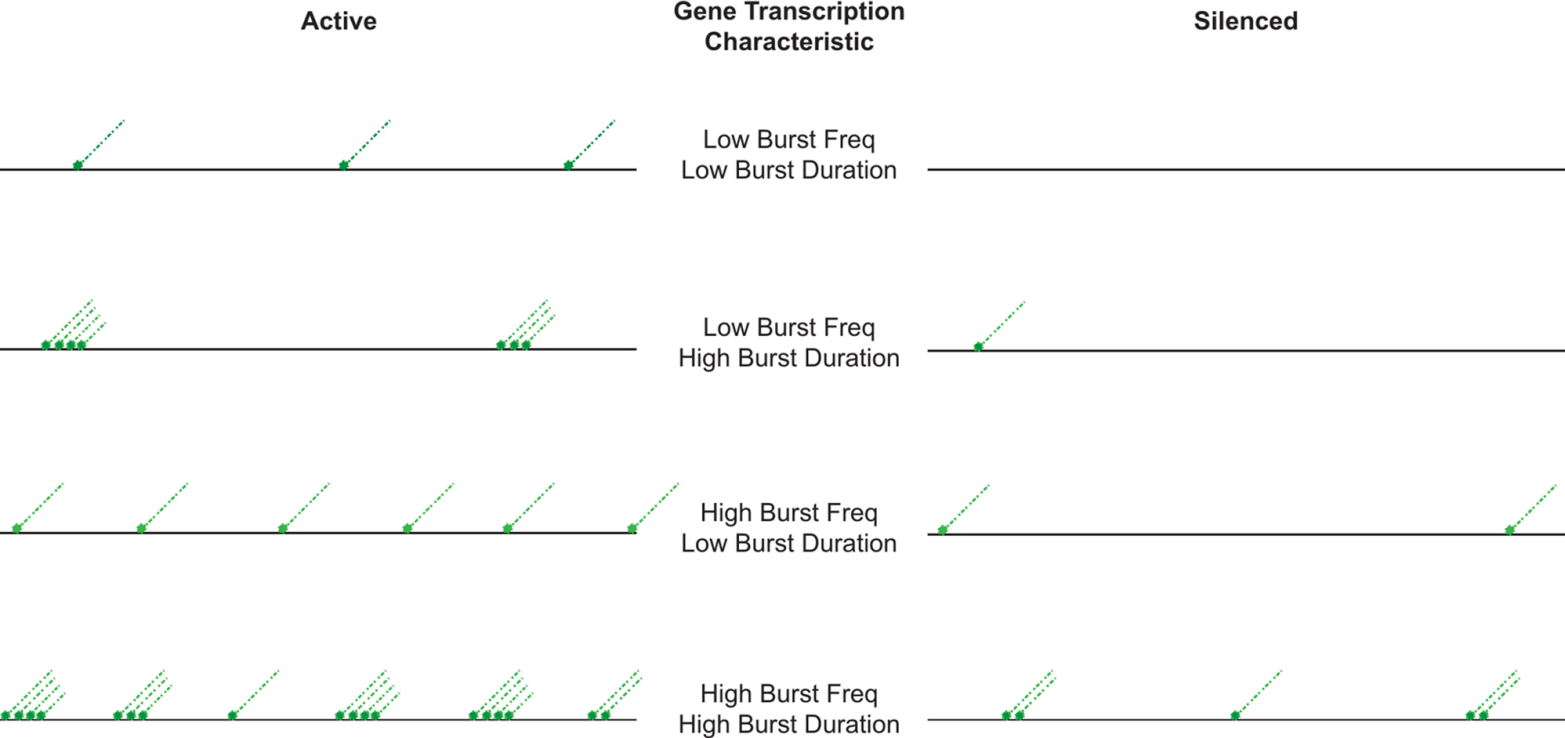
A schematic of different transcription initiation frequencies and burst durations. Green trails represent transcription events. The number and clustering of transcription trails reflect bursting frequency and duration. X-axis reflects time

The transient interactions between TFs and DNA result in functional consequences. There is a temporal coupling between binding events at the enhancer and the promoter but the relationship is not simple [37–39]. TFs bind their sites transiently in chromatin and recruit chromatin modifiers and remodellers to stabilize open sites [40]. Stable TF and mediator binding then create temporal windows during which information is transferred, possibly through allostery, to the GTFs bound to the core promoter resulting in transcription initiation and convoys of RNA polymerase [41]. The length of a transcription burst, how often a burst of transcription occurs and how many transcripts are produced in a burst are regulated. In broad terms, the frequency of TF binding to an enhancer affects transcription burst frequency while core promoter sequences affect burst duration by modulating residence times of GTFs [37, 42, 43]. There are different individual binding timescales that collectively determine bursting. These include TF binding and unbinding, mediator dynamics, chromatin modifier and remodeller binding dynamics, nucleosome cycling and GTF binding and release amongst others [39]. Furthermore, the different steps do not have identical reaction rates and this coupled with fluctuations in concentrations of factors generates transcription initiation events that fluctuate. Some genes like housekeeping genes are highly transcribed, more or less continuously, while others are rarely transcribed or transcribed only under inducing conditions in bursts [44]. Within this framework, transcriptional silencing could be visualized as the ultimate endpoint of burst regulation resulting in a completely inactive state that dampens some or all of these steps and persists across multiple cell cycles (Figs. 1 and 7) but questions remain regarding the identity of the specific step/s and the mechanism by which silencing is affected.

Current data suggest that a simple thermodynamic equilibrium framework is not sufficient to fully describe gene regulation [45, 46] and nucleosome dynamics play an important role in this process [37, 47]. The affinity of a nucleosome for a site is typically greater than the affinity of the TF for that site [27, 48, 49]. The DNA wrapped around nucleosomes also constantly breathes via thermal motion creating temporal windows of accessibility and inaccessibility for TF binding [40, 50–53].

There is a high degree of nucleosome organization at regulatory regions with well-positioned + 1 and − 1 nucleosomes flanking a nucleosome depleted region (NDR). The NDR encompasses the UAS enhancer and core promoter [54–57] and the NDR is actively generated and maintained by ATP expending chromatin remodelers aided by post translational modifications of histones [6, 31, 46, 49, 58–64]. The precise location of the + 1-nucleosome adjacent to the NDR influences transcription initiation [65] and is likely an important arbiter of transcription bursting. If the rate of sliding/removal of nucleosomes or the rate of unwrapping of the DNA from the nucleosome surface is decreased, then nucleosomes hinder accessibility of sites even when TFs are present and the probability of transcription corresponds in part to the rate of TF binding site exposure in nucleosomes and it is likely that silencing affects these rates.

**Fig. 2:**
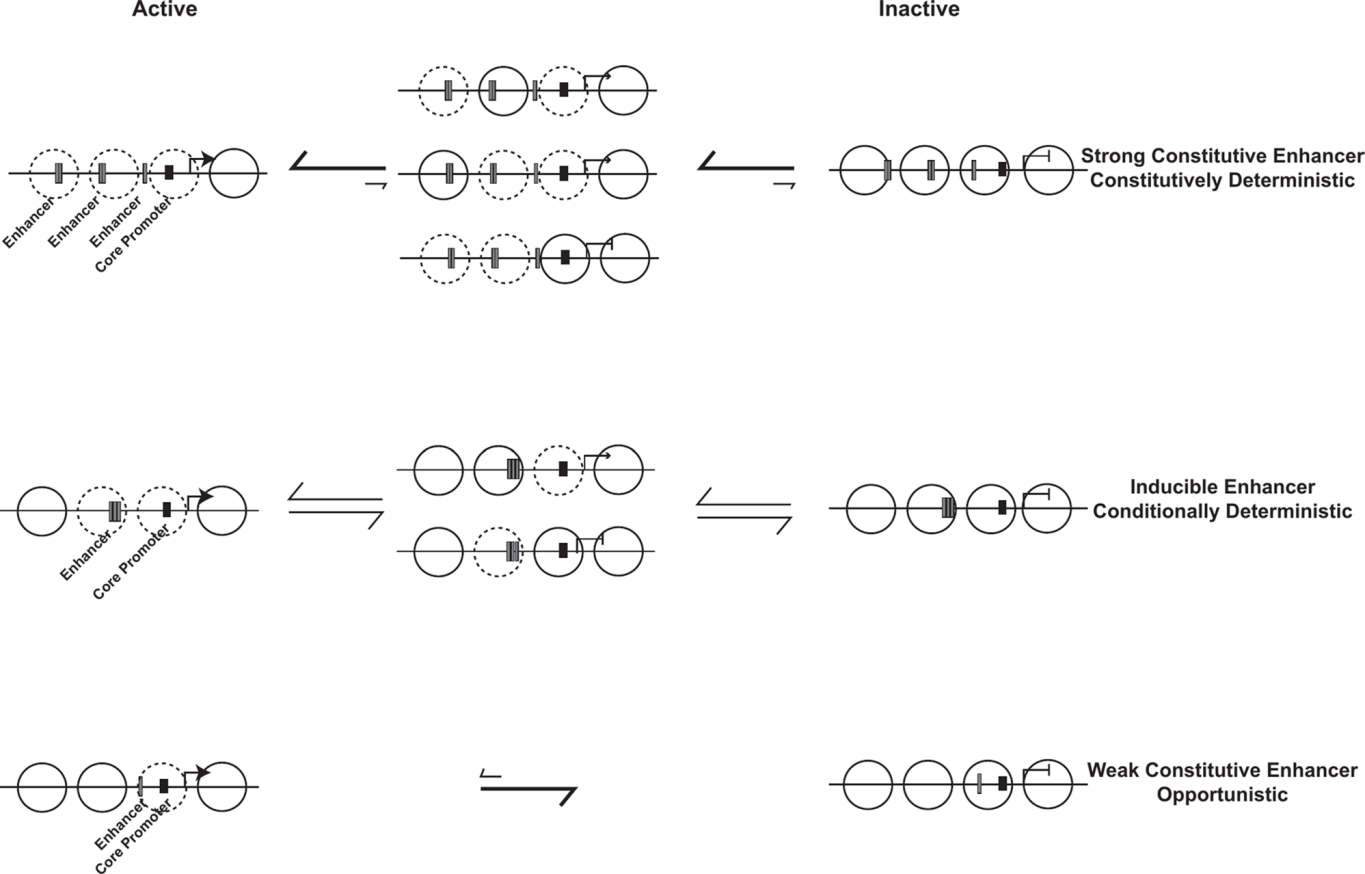
Schematic of nucleosomes cycling through different active and repressed chromatin configurations for different classes of UAS enhancers. Full circles indicate nucleosome presence, dashed circles indicate nucleosome depletion. Core promoters are shown as black rectangles while gray rectangles highlight TF binding sites in UAS enhancers

In a population of cells there exist different nucleosome configurations over the regulatory regions of genes; some that are conducive for transcription and others not (Fig. 2). Surprisingly, all possible nucleosome configurations are observed in the population regardless of the gene’s average expression state [47, 66]. The gene cycles through different nucleosome configurations and dwells for different lengths of time in each configuration. The key difference is the frequency of the different nucleosome configurations. The fully nucleosomal configuration is more often found under repressing conditions (right panels in Fig. 2) while very few gene regulatory regions are fully nucleosomal under activating conditions (left panels in Fig. 2). TFs and chromatin remodelers regulate transcription by changing the probability distribution of different nucleosomal configurations at regulatory sites and the Sir proteins may function to alter the frequency of these probability distributions during silencing.

Some genes such as housekeeping genes are regulated in a deterministic (less noisy) manner while others utilize opportunistic mechanisms [67]. Some TFs called general regulatory factors function deterministically- they are constitutively present at high concentrations and recruit chromatin remodellers to reposition and mobilize nucleosomes (see top panel in Fig. 2). In a population of cells, they increase the likelihood of chromatin configurations that favour high levels of transcription burst frequency and duration (see [68] for a detailed description). Inducible genes are regulated by TFs whose biochemical characteristics and concentration in the nucleus change under inducing conditions and these TFs then alter the nucleosome configurations using varied mechanisms [5, 6] (middle panel in Fig. 2). The end point of this is a change in the probability distribution of active versus inactive nucleosome configurations leading to sustained bursts of transcription under inducing conditions [47]. The final class of genes are entirely opportunistic- they may not rely on TFs [67] but on stochastic removal of nucleosomes from core promoters by free floating chromatin remodellers and histone modifiers allowing PIC formation and these genes are the weakest in terms of transcription output (bottom panel in Fig. 2). Thus, each class of genes has different probabilistic expression profiles dependent on the frequency of the different nucleosomal architectures that are generated by chromatin remodellers and histone modifiers and the responses of these genes to silencing factors is correspondingly different [69].

## Silencer activity

Silencers are essential for silencing [70] and possess a robust NDR bound by combinations of multi-functional DNA binding proteins ORC, Rap1, Abf1 and Sum1 [71, 72]. A signature function of silencers is the “recruitment” of the Sir2, Sir3, Sir4 proteins to the silenced domain [73–78] and possibly in channeling chromatin remodelers to generate evenly spaced nucleosomes thus creating a directionality in the spread of silencing [79–81]. Once Sir2 and Sir4 are recruited to the silencers, Sir2 then deacetylates histones in adjacent nucleosomes enabling Sir3 and Sir4 to bind to the hypoacetylated evenly positioned nucleosomes [76, 77, 82–84]. Repetition of this process leads to Sir protein spreading creating a silenced domain [9, 13, 71].

We understand the function of the silencers but several questions persist regarding their mode of action. How the specific configuration of sites and proteins at silencers result in silencing as opposed to gene activation remains unclear. Like models for enhancer function, do silencers and their associated proteins function like billboards performing distinct, independent roles or do they function by forming a large complex analogous to the enhanceosome [85, 86]? Whether silencer bound proteins are regulated during silencing is also not known [87, 88] and whether their binding to the silencer depends upon cofactors such as Sir1, chromatin modifying and remodeling factors is also not known.

Like enhancers, different silencers have varying strengths [89, 90]. This is likely due to the differences in the binding parameters of silencer bound proteins with one another, with DNA and with the Sir proteins. While silencer bound proteins are undoubtedly subject to the laws of thermodynamics, surprisingly little is known about the affinities, residence times and cooperativity of these proteins at silencers. The binding of ORC to the silencers appears to be stronger than its interaction with euchromatic origins of replication [91] while measurements of residence time of Rap1 show faster turnover at silenced telomeres compared to sites at enhancers [92]. Thus, while additional research is necessary, one model is that differences in thermodynamic and kinetic parameters result in differences in the amount of Sir proteins retained/recruited in the vicinity of the silencers leading to differences in silencer strengths.

In vertebrates, DNA sequence elements functioning as silencers have not been identified for HP1 or polycomb mediated silencing but silencer like elements have been annotated and analyzed in *S. pombe* [93, 94] and *Drosophila* [95]. In the instances where silencers have been demarcated, the functions ascribed to these DNA elements are analogous to the roles ascribed to silencers in S. *cerevisiae* (reviewed in [15, 96–101]). Like Sir mediated silencing, nucleation and spread of heterochromatin in other eukaryotes involves targeting methylases to methylate histones in nucleosomes followed by binding of the methylases and repressor proteins to the methylated histone leading to the spread of silenced chromatin [102, 103].

## The nature of silenced chromatin domains

**Fig. 3:**
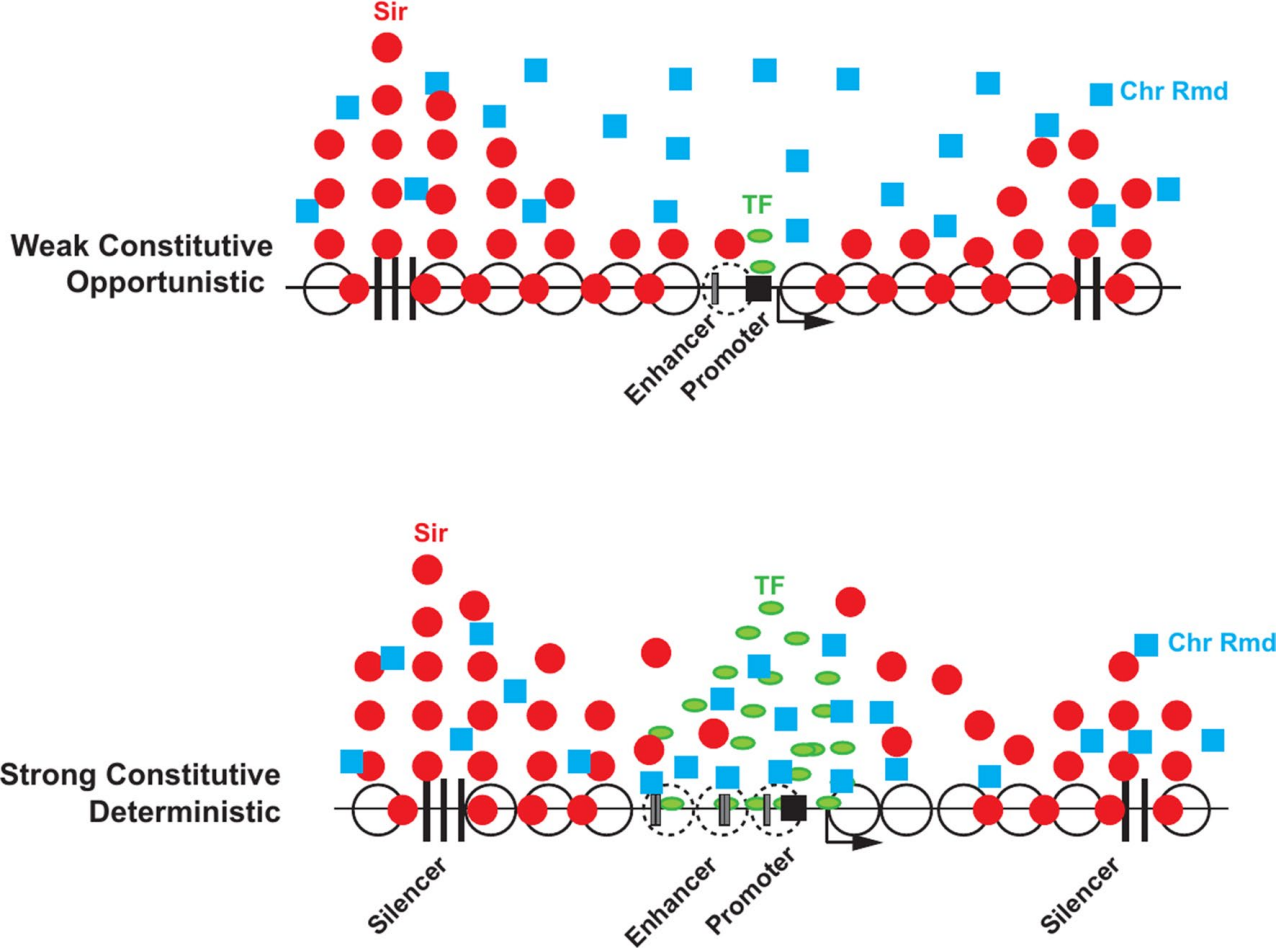
Schematic of the distribution of Sir proteins and TFs at silenced loci with different classes of UAS enhancer/core promoters. Red dots represent Sir proteins and peaks of distribution, green ovals represent TFs, blue squares indicate free-floating chromatin remodellers. Vertical black bars represent silencers and gray boxes represent enhancers and black boxes represent promoters

Chromatin plays a critical role in gene silencing. One defining property of silenced chromatin is that it is marked by hypoacetylated histones H3 and H4 that are necessary for Sir protein binding (reviewed in [13]). Consistent with this, ChIP-seq mapping of H4K16 acetylation reveals uniformly hypoacetylated histones across entire silenced domains [104–106]. The silenced domain is also marked by evenly spaced nucleosomes with long linker DNAs. This nucleosome configuration is silencer dependent and disrupted in Sir mutants suggesting that Sir-nucleosome interactions play a role in organizing/stabilizing nucleosome configurations [81, 107, 108]. Surprisingly, the regulatory regions of silenced genes have accessible NDRs [109, 110]. Furthermore, ChIP indicate that the Sir proteins are highly enriched at the silencers with reduced levels at the enhancers/promoters of the silenced genes [104–106, 111] (Fig. 3). Alternative methods mapping dynamic interactions also show localized peaks of Sir proteins at silencers [110]. Consonant with these are studies which show highly localized peaks of Sir protein at sub-telomeric sites with flanking nucleosomes bound by Abf1 and Reb1 creating a trinucleosome repressed domain [67, 112] consistent with early data showing discontinuous silencing domains [113–115] though it is unclear if this architecture is present in all cells or is a population average image derived from heterogeneity of binding.

Data show that the Sir proteins are mobile in the nucleus [70, 82, 116, 117] and constantly exchange between sites. The peaks of Sir proteins at the silencers suggest that their residence time at silencers is likely to be high while the lower levels within the silenced domain suggest that their binding to nucleosomes is more transient. This raises the question of whether stable Sir protein binding to nucleosomes is necessary to block transcription or whether deacetylation of histones and/or preventing movement of nucleosomes across the entire domain is the key driver in silencing and illustrates the importance of considering binding kinetics. While the binding affinities of Sir3 for acetylated and unacetylated nucleosomes are known [118], we do not have information on affinities of the other Sir proteins or the residence time of the Sir proteins bound to chromatin. To gain a more granular picture, it is necessary to compare residence times and binding constants of Sir proteins at silencers versus nucleosomes as well as the turnover rates of histone modifications.

The end point of Sir protein binding to nucleosomes is the inhibition of transcription. A classical study measuring accessibility of chromatin to a DAM methylase enzyme demonstrated that the Sir proteins reduced access to DNA [119] though a recent study failed to observe significant reduction in accessibility at silenced chromatin to other probes [120]. In vitro studies of Sir bound chromatin also highlight reduced accessibility to various enzymatic probes [118, 121, 122] (see [123] for detailed discussion on chromatin accessibility assays). While these studies showed reduced accessibility but not inaccessibility, they highlight the dynamic but restrictive nature of silenced chromatin. It should be noted that most of the in vitro studies were performed in the absence of active chromatin modifying and remodeling complexes under conditions that favor stable binding of Sir proteins to nucleosomes.

Based on these studies simple steric hindrance models were proposed where Sir proteins bound to key nucleosomes stereospecifically blocked the binding of TFs or GTFs to regulatory sequences. Consistent with these models, ChIP mapping experiments with different reporter genes showed that silenced chromatin restricts different transcription proteins from binding at different enhancers and promoters [124–128].

The question then is how the Sir proteins block multiple factors and processes via a single binding mode. The presence of an accessible NDR at the regulatory regions of silenced genes, the cycling of different chromatin configurations at regulatory sites, the mobility of Sir proteins, and the non-uniform distribution of the Sir proteins across the silenced domain raise questions regarding these simple steric hindrance models for silencing. Gene activation is a multi-step dynamic process and different gene regulatory elements and their cognate TFs use different cofactors in different temporal order to generate a PIC and initiate transcription [5, 6]. It is likely that the Sir proteins alter specific rate-limiting steps in this process to mediate silencing. One possibility is that the Sir proteins alter a common early step while being agnostic to the diversity of downstream effector proteins. One early common step in gene activation is the mobilization of nucleosomes from regulatory sequences prior to transcription. Sir proteins could directly or indirectly affect the rate of sliding and/or removal of nucleosomes from regulatory sites. This would alter the probability distribution of active versus repressed nucleosomal configurations which would then have downstream effects on TF binding, PIC formation or post initiation events. While replication-independent histone exchange is infrequent and affects only 1 to 10% of nucleosomes [64], there is a correlation between the level of exchange and RNA polymerase levels [129]. Consistent with this, silenced heterochromatic genes have lower histone exchange compared to active euchromatic genes [130] though the promoters of silenced genes have increased histone turnover compared to the coding regions of these genes (which might help explain the NDR at these loci). In addition, an in vitro study suggests that the Swi/Snf chromatin remodeller interacts with Sir3 and this interaction is necessary for the eviction of Sir3 from reconstituted heterochromatin [131]. A recent study also showed that reducing nucleosome density and increasing the degree of freedom for nucleosome movement destabilized the silenced state [132]. While in vivo data with remodeller mutants could be due to indirect pleiotropic effects a recent analysis of chromatin remodeler mutants with silenced reporter genes [69] was consistent with and as such supportive of this model.

It will be interesting to know the frequency distributions of different nucleosomal configurations when a gene is active versus silent as well as the precise positioning of nucleosomes and the role of chromatin remodellers in this process. Knowing the binding affinities and residence time of the GTF/TFs at silenced enhancers and promoters would also be informative and allow the testing of different models.

This model builds on pioneering studies in other eukaryotes showing that polycomb and trithorax complexes colocalize [133] and facultative heterochromatin mediated repression is mediated in part by inhibiting chromatin remodellers and histone acetylases [15, 31, 134–138]. Similar mechanisms have also been shown to operate for constitutive HP1 containing heterochromatin. The turnover dynamics of nucleosomes are conserved across sites in *S. pombe* and *Drosophila* [139–142] raising the possibility that different silencing proteins (and different chromatin modifications) might utilize similar mechanisms to mediate silencing in all eukaryotes.

## Sir protein structures and models for gene silencing

**Fig. 4:**
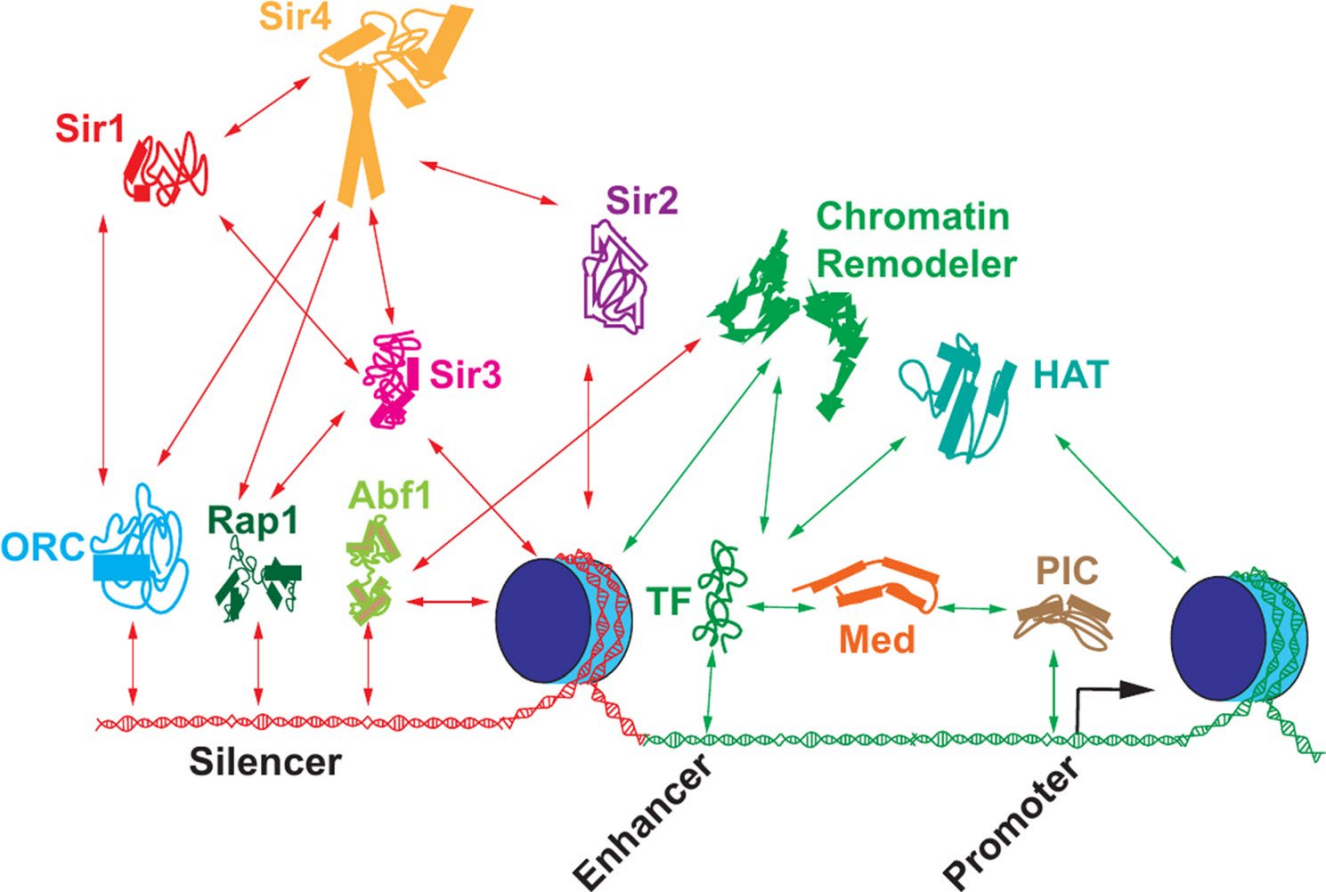
Schematic of the numerous multivalent interactions between the different factors involved in transcriptional silencing. Red arrows depict interactions involved in silencing while green arrows depict interactions involved in activation

The next question is how the Sir proteins alter nucleosome dynamics. Sir proteins bind nucleosomes and each other and the nature of this stereospecific binding is central to silencing. The web of multivalent interactions (Fig. 4) suggests coordinated and/or sequential interactions between Sir proteins and their partners in silencing.

**Fig. 5:**
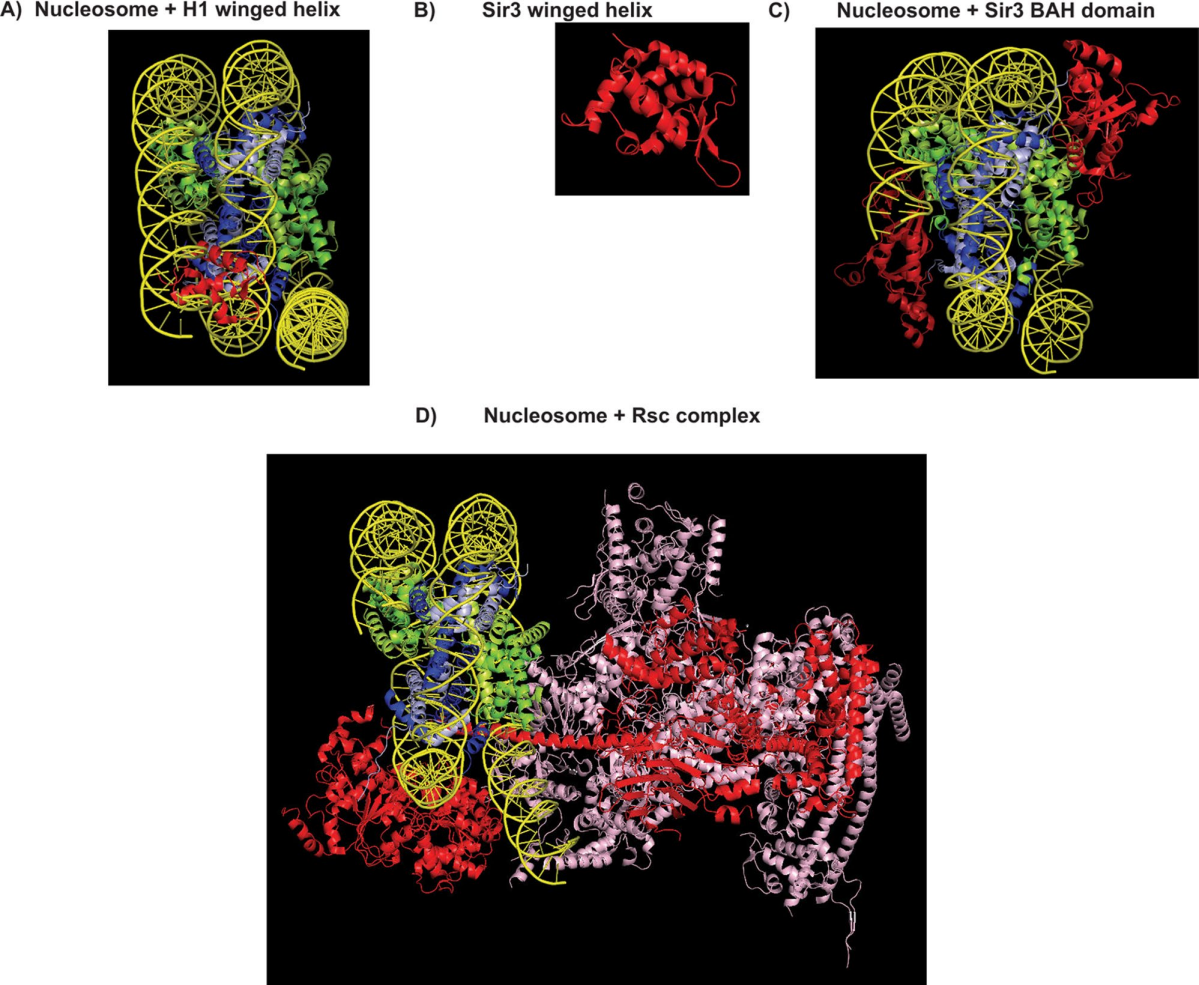
Molecular structures of various nucleosome bound and free protein complexes. Histones H3 and H4 are coloured dark and light blue while the histones H2A and H2B are coloured dark and light green. **A** Structure of a nucleosome bound by the winged helix (in red) of histone H1. **B** Structure of the winged helix domain of Sir3. **C** Structure of a nucleosome bound by the Sir3 BAH domain (in red). **D** Structure of a nucleosome bound by the RSC complex (BAH domain containing subunits of RSC are shown in red)

The structure of several Sir protein domains has been solved though the structure of the holocomplex has not yet been determined [122, 143–152]. These structural studies highlight possibilities by which Sir binding to nucleosomes could affect chromatin configurations and silencing. Sir3 contains a winged helix domain which is important for Sir3-Sir3 interactions that are necessary for its binding to nucleosomes [122]. Structural studies on the H1 winged helix-nucleosome interactions are suggestive of how Sir protein binding might play a role in nucleosome mediated silencing [153, 154]. Analogous to the H1-nucleosome structures, the binding of the Sir3 winged helix to the nucleosome and the induced clamping of the H4 tail with intranucleosomal DNA could reduce nucleosome mobility thus indirectly restricting access of the transcription machinery (Fig. 5). In this model Sir protein binding would raise the energetic barrier for the opening of chromatin thus inhibiting transcription. Second, Sir2 mediated histone deacetylation is likely to strengthen histone-DNA interactions thus reducing breathing of nucleosomal DNA [156] and thus reducing the rate of chromatin opening. The reduced breathing of the clamped nucleosomal DNA would close a window of opportunity for TF binding. Third, unacetylated tails have a reduced affinity for chromatin remodeler binding thus discouraging nucleosome movement [157]. Sir protein binding to nucleosomal linker DNA [110] results in even spacing and positioning of nucleosomes with long linkers [81, 107, 108] which would likely help in nucleosome stacking and interdigitation of the chromatin filament [158] thus enabling internucleosomal interactions and chromatin compaction [159–161] which could reduce TF access to DNA.

There are two classes of BAH domains in yeast- Sir3 like and RSC like [155] and these domains are involved in protein-nucleosome interactions. Structure determinations show that Rsc binds nucleosomes at sites close to those occupied by the Sir3 BAH domain [148, 155, 157, 162–164] (Fig. 5). A higher k_off_ for Rsc compared to Sir3 (whose k_off_ is currently unknown) could therefore favor a Sir3-nucleosome bound state at genes undergoing silencing thus highlighting another molecular mechanism that could influence chromatin remodeler mediated nucleosome mobility.

BAH domains are also necessary for H3K27me3 recognition in nucleosomes by polycomb proteins [165] while interactions between HP1 and histone H1 have been shown to function in HP1 mediated silencing [18]. All these models are speculative and in need to testing. Thus, while the structures of Sir protein domains have been informative, the structure of a Sir holocomplex with oligonucleosomes would be illuminating. Biochemical reconstitutions of silenced chromatin in the presence of silencers, sub-saturating levels of the Sir proteins and chromatin with different repeat lengths would also be informative.

## The role of Sir protein compartments in silencing

Silenced domains localize to the nuclear periphery and are anchored by numerous factors (reviewed in [166–171]) leading to the tethering of chromatin fibers and the clustering of these domains. While differential properties of chromatin have been suggested to promote phase separation and compartmentalization of the nucleoplasm [172], there is as yet no evidence of Sir proteins forming liquid droplets. Clustering of domains creates silencing compartments enriched for Sir proteins (reviewed in [12, 168, 169, 173, 174]) and this is important for transcription repression [175]. There are approximately 57,000 nucleosomes in yeast [57] and roughly 10% of these are hypoacetylated [176]. Given the sizes of silenced regions, one can estimate that ~ 2/3 of the hypoacetylated nucleosomes are present at silenced domains. High-throughput studies suggest that there are ~ 1400 molecules of Sir3 and Sir4 protein in a typical yeast cell [177] which is significantly fewer than the number of unacetylated nucleosomes. Consistent with this, analysis has shown that Sir proteins are a limiting component of stable silencing [83, 90, 156, 178, 179].

**Fig. 6:**
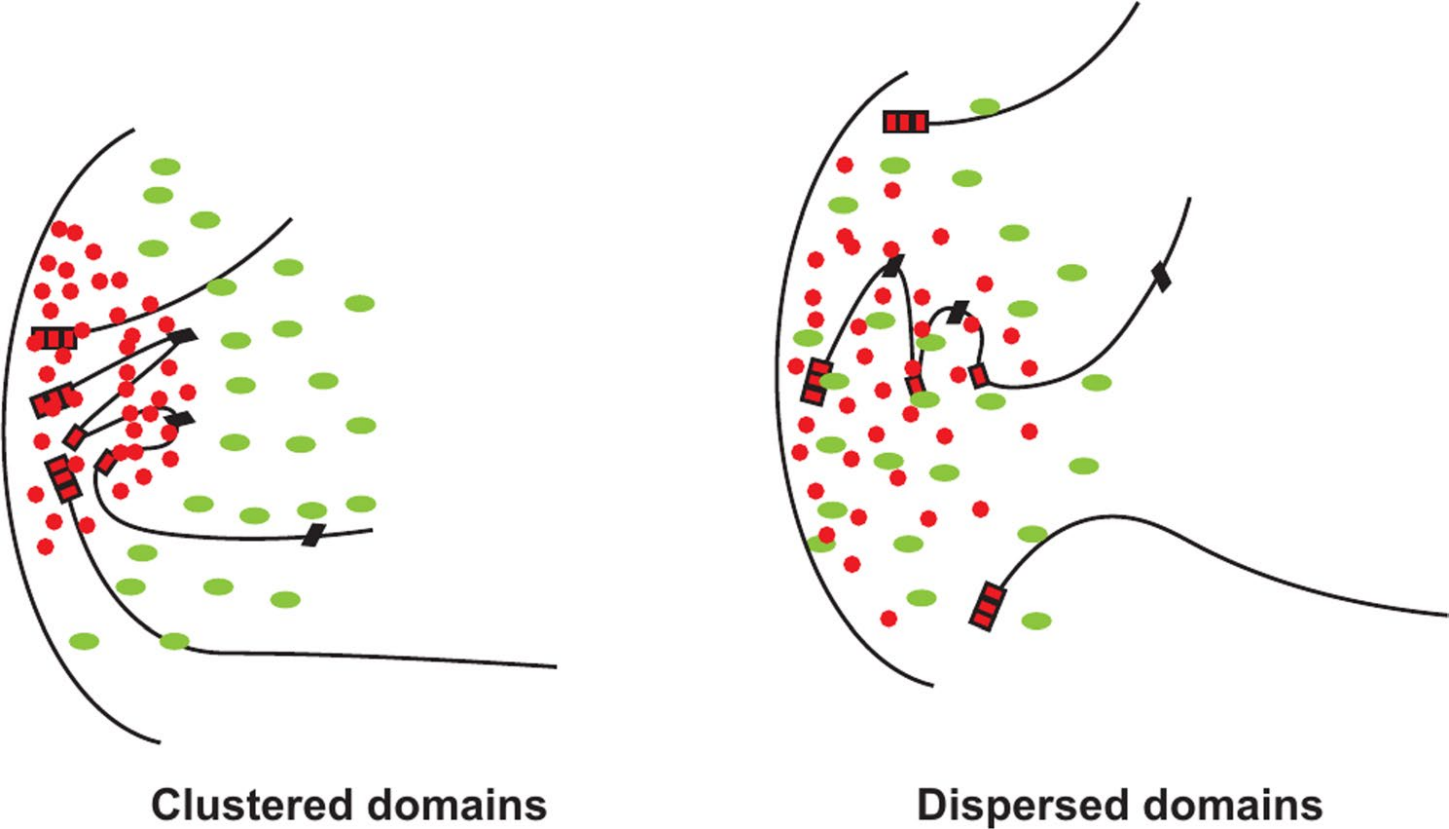
Schematic of the effects of clustering of the silenced chromatin domains. Red dots represent Sir proteins, green ovals represent TFs, rectangles represent silencers. Trapezoids represent enhancers

Clustering of silenced domains would aid in trapping of Sir proteins amongst the clustered loci (pinball effect) creating a compartment with elevated concentrations of Sir proteins which would then in turn influence binding equilibria [111, 169, 180] (Fig. 6). It is informative to consider a hypothetical scenario. If one Sir protein is present in a yeast nucleus with a diameter of 2 μm (volume ~ 4 μm [3] or 4 fl.), this is equal to a concentration of ~ 2 nM. If the nucleus is compartmentalized and if one were to restrict one Sir protein to a compartment that is 10% of the diameter of the typical nucleus, the Sir protein concentration would increase nearly 1000-fold providing a powerful way to increase the effective concentration. Recent data show that one of the earliest steps in the establishment of silencing involves the perinuclear anchoring of silenced loci that aids in the accumulation of Sir proteins which reinforced anchoring culminating in a self-reinforcing loop in silencing [181].

Clustering could therefore alter thermodynamic parameters and Sir concentration is likely to be one important parameter. Measurements in mammalian cells show an approximately two-fold difference in nucleosome density between euchromatin and heterochromatin [182] but this leads to subtle effects on molecular diffusion and movement of transcription factors [183, 184] and it is therefore unlikely that silencing can be solely described by these values alone especially given the non-equilibrium nature of gene regulation and the relative immobility of tethered chromatin fibers.

Alterations in concentration will also not alter stereospecific interactions between Sir proteins and nucleosomes but would create a “circe” effect [180]. Increased Sir concentrations would reduce the search times required for Sir proteins to find, deacetylate and bind unacetylated nucleosomes (k_on_) and would alter the probability distribution of hypoacetylated Sir bound nucleosomes. Nucleosomes that localize within clouds of high Sir protein concentration would have a higher probability of becoming and remaining hypoacetylated. The concentration dependent macromolecular crowding might also alter TF search times and K_on_ thus hindering TF and chromatin remodeler access [183–188]. This is consistent with studies where merely tethering a locus to the nuclear periphery results in Sir mediated repression of reporter genes [189, 190]. The increased concentration may aid in cooperativity between Sir proteins though no Hill coefficient measurements have been reported for silencing components. While clustering should not alter the residence times of the Sir proteins bound to nucleosomes, the higher concentration of free-floating Sir proteins in the nucleoplasm might also facilitate dissociation of TFs from UAS enhancers thus indirectly increasing the effectiveness of silencing.

The clustering of silenced domains also brings distant sequences into close three-dimensional proximity [111, 191, 192] and this helps in Sir protein dependent long-range internucleosomal interactions [122, 159, 193]. Recent analysis with specific mutant alleles showed that Sir3-Sir3 internucleosomal interactions promoted long-range chromatin contacts most likely via the winged helix domain of Sir3 [161]. The three-dimensional web of interactions between silencers, Sir proteins and nucleosomes would create a dynamic 3D structure with a self-enforcing loop that stabilizes the silenced state [194, 195].

The importance of subnuclear compartments in silencing is highly conserved across eukaryotes. Polycomb and Swi6 mediated compaction and phase separation aids in H3K27me3 and H3K9me3 mediated facultative and constitutive heterochromatin [141, 196–203] highlighting some commonalities in processes between different heterochromatic systems.

## Differential susceptibility of enhancers and core promoters to silencing

Silencing has traditionally been considered a gene agnostic phenomenon that could silence most genes. However, recent analysis of native genes at silenced loci using mRNA-Seq showed that only a few genes at native telomeres are silenced while many others resist/escape silencing [104–106]. Detailed analysis of discontinuous silencing at native telomeres showed that a dynamic competition between TFs and histone deacetylation by Sir2 determines the extent of silencing at these sites [112]. Recent studies measuring silencing of a set of gene regulatory elements using multiple reporter assays including directly visualizing transcription in single cells also showed that Sir proteins stably silence only weak and uninduced stress response regulatory elements but are unable to stably repress strong housekeeping gene regulatory elements and they do so by altering transcription bursting [69].

Chromatin configurations at regulatory sites constantly cycle between open and closed states. The enhancers that are silenced have weak or non-existent TF binding sites with low burst frequency and duration and thus succumb to silencing - monostable repression. For activation, these genes utilize opportunistic mechanisms, where chance opening of the regulatory sequences by nucleosome sliding/movement by free floating chromatin remodellers allows for the transcription machinery to initiate transcription. The presence of Sir proteins bound to nucleosomes would reduce the mobility of nucleosomes and in a population of cells these genes would predominantly be packaged in chromatin configurations that disfavor transcription thus reducing the probability of gene activation (Fig. 2). In this statistical scenario, the Sir proteins would function not by increased condensation of chromatin or physically blocking a factor from binding but by reducing the rate of chromatin opening.

Strong housekeeping genes resist silencing and remain active- monostable activation. These strong constitutively active regulatory elements bound by general regulatory TFs, with their attendant biochemical properties, exhibit high burst frequency and duration and remain active [69]. The ability of these genes to resist silencing (monostable activation) is likely determined by varied criteria such as the type of TFs, the number of binding sites for the TFs, the concentrations and the binding characteristics of these proteins and their ability to recruit chromatin remodelers and modifying enzymes [68, 112]. These factors together likely affect the probability distribution of active versus inactive chromatin configurations to favor the accessible chromatin states. This may also be the underlying reason why heterochromatin barrier insulators are populated by such sequences [115, 204–206].

Under certain circumstances, bistable expression states arise for genes undergoing silencing [207–209]. Bimodal expression patterns in genetically identical cells arise from direct competition between TFs and repressors for binding to the same site [210]. Bimodal expression states become bistable (expression states that persist through cell division) due to either positive feedback or double negative feedback coupled with cooperativity (non-linearity) [211, 212]. Bistable expression states arise at silenced loci under specific conditions- when mutations in the silencers weaken silencing, when a relatively strong activator binds the UAS enhancer of a reporter gene or when a silenced domain is transposed to a euchromatic site in the nucleus where Sir protein concentrations are lower [69, 70, 207–209].

**Fig. 7:**
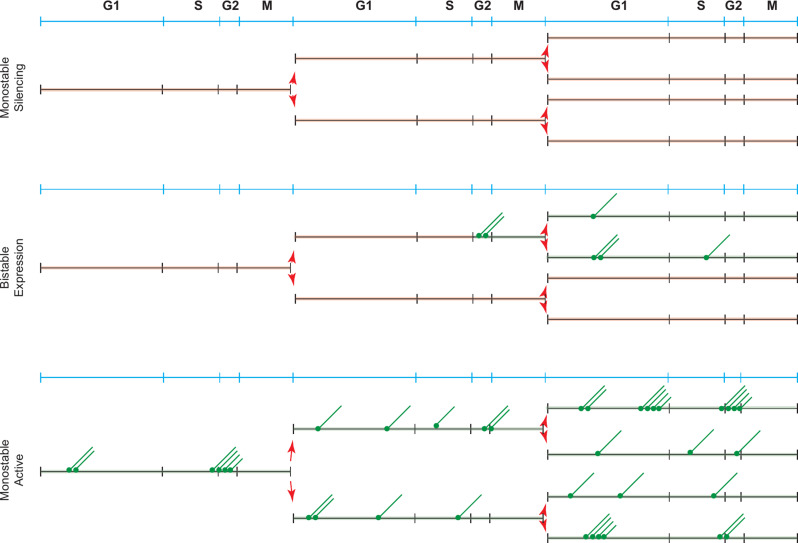
Relationship between stability of silencing and the inheritance of expression states through the cell cycle. Red lines highlight silenced state while the green lines reflect the active state. Green trails indicate transcription. Blue line represents phases of the cell cycle

In the context of bistable expression states, one question that naturally arises is how the active state persists in competition with silencing (and vice versa) given that activation is a multi-step probabilistic process where each step is naturally inefficient, stochastic and transient (bursty) [37, 213, 214]. One possibility the active state is stably maintained is because the establishment of the silenced state is an inherently slower process, compared to activation, and requires an extended period of transcription inactivity to fully form [181, 215]. A gene that is repeatedly activated (even for short bursts) would prevent a stable silent state from forming (Fig. 7).

**Fig. 8:**
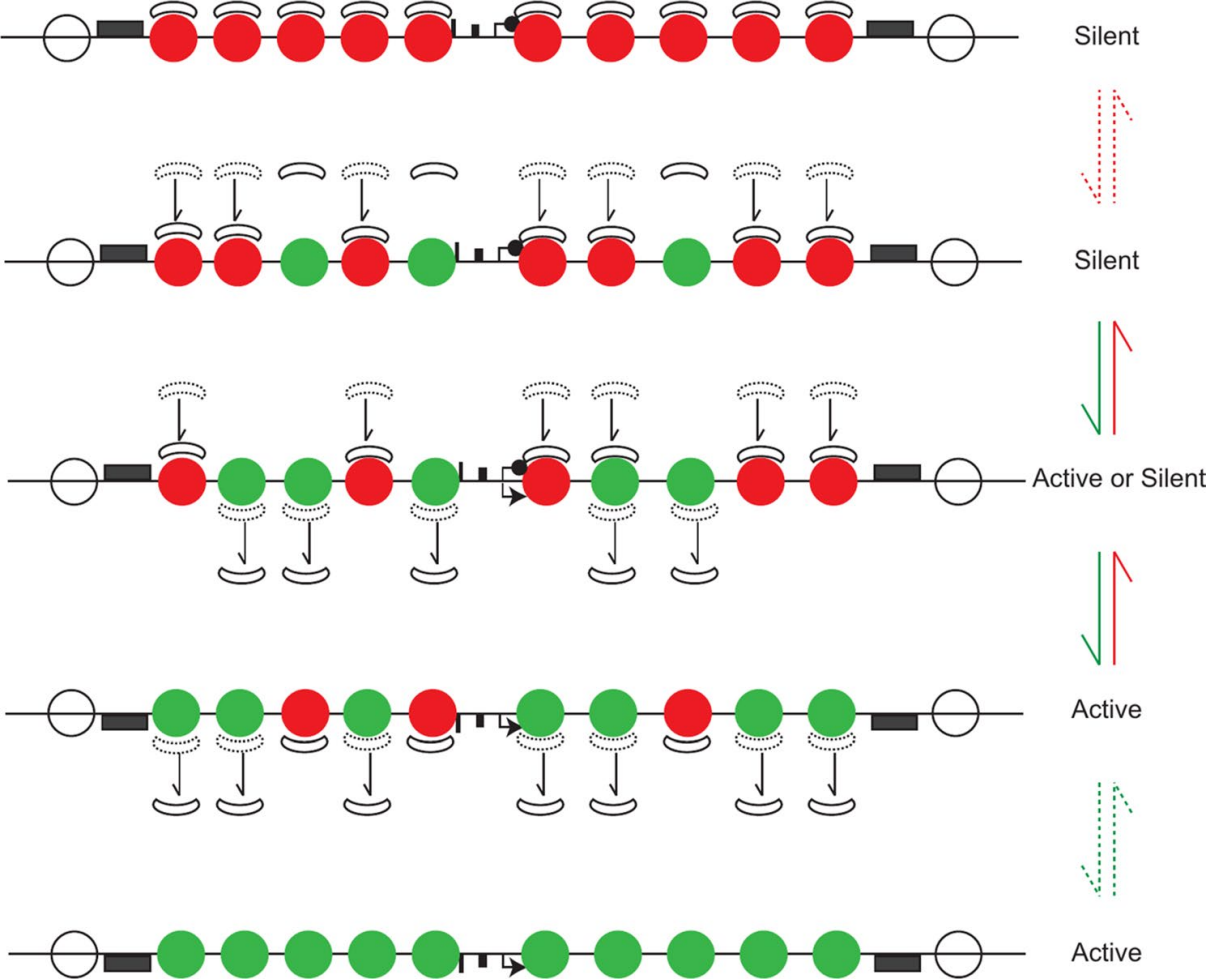
Hysteresis in gene silencing. An active domain requires deacetylation of 75% of the nucleosomes to become silenced. A silent domain requires acetylation of 75% or more nucleosomes to lose silencing. Red circles denote unacetylated histones and green circles denote acetylated histones. Moon shapes denote Sir proteins; gray rectangles are silencers and black rectangles are enhancers and promoters

Another reason for the observed stability is that the silenced domains exhibit hysteresis (see [216–218] for a description of hysteresis). The response of the silenced domain to changes in histone acetylation depends on whether the system is in its off or its on state and Sir2 enzymatic activity is the driver of the hysteresis effect [219, 220]. Recent work shows that if the locus is silent, then 75% of nucleosomes need to become acetylated (acquire an acetyl mimic histone H4 (K16Q)) before the system loses silencing [90]. If the locus is active (because of the presence of the histone H4K16Q allele), then greater than 75% of nucleosomes must acquire unacetylated histones (wild-type H4K16) for an extended period of time to establish silencing (Ken Wu and RTK unpublished results). This Sir2 and histone acetylation mediated hysteresis effect could therefore also contribute towards the observed stability of the silenced state (Fig. 8).

The observations that Sir proteins can only stably silence a subset of genes is also reminiscent of earlier observations with polycomb proteins [221]. These proteins were shown to only stably silence specific promoters and most importantly the primary function of these proteins was the stable maintenance of the inactive state after transcription of the gene had ceased (reviewed in [15]). Recent studies with synthetic silencing systems mirror these conclusions [222, 223].

## Domain wide regulation of stable heritable silencing

One defining property of silencing (albeit of weakly transcribing genes) is stable maintenance of the silenced state and the high fidelity of inheritance of this state once it is established [224, 225]. The silent domain is a stable structure created by Sir proteins in constant flux. Measurements of the stability of silencing showed that in wild type cells, silencing at *HML* is stochastically lost in one out of every 1000 cells with a similar value at *HMR* [224].

Interestingly, once the silenced domain is established it can tolerate significant fluctuations in the levels of Sir proteins and nucleosomal acetylation without loss of silencing. Silencing is only lost when approximately 75% of the nucleosomes across the silenced domain acquire H4K16 acetyl like marks [90]. While it is possible that the deacetylation of a single nucleosome is important in silencing, these data argue against this. Interestingly, simple calculations based on these data highlight the possibility that a rather small (less than two-fold) reduction in the ability of acetyltransferases to acetylate a nucleosome, spread across a domain of 20 nucleosomes, might be enough to establish a silent domain [90]. What these data also suggest is that the nucleosomes across the clustered silent domains function together where each nucleosome bound by Sir proteins acts as weak point silencer helping maintain transient elevated local concentrations of Sir proteins. The many multivalent interactions between Sir proteins and nucleosomes would create a 3D-mesh of interactions (Fig. 4) and each interaction would create a reinforcing feedback loop where Sir2 deacetylation of histones would facilitate Sir3 binding which in turn could facilitate interactions with Sir4 and its interacting partner Sir2 [211, 212]. Once a critical mass of Sir proteins bound to nucleosomes is achieved, the system becomes self-sustaining and stable. Thus, while the individual Sir-nucleosomes interactions are weak and therefore transient, the overall system would exhibit stability so long as multiple nucleosomal sites remain bound by Sir proteins for sufficiently long periods of time. In this system the transient removal of Sir proteins from a single nucleosome, even one over a key regulatory sequence would be unlikely to initiate or allow persistent and sustained gene activity.

In conclusion, silencing is a weak form of repression and there are numerous factors that together result in a stable silenced state. Silenced chromatin only efficiently represses weak enhancers and promoters. The silencer and silencer bound proteins are necessary for the efficient maintenance of the silent state. They function by increasing the local concentration of the Sir proteins. The clustering of silenced loci is important in this process. The data suggest that the silenced state depends on local Sir-nucleosome interactions as well as a domain wide web of interactions analogous to what has been proposed for phase separation in gene activation [26]. The stability of the final state is likely influenced by numerous factors such as the concentration of the Sir proteins, transcription activators, architecture of gene enhancers and promoters, histone modifying enzymes, the positioning and dynamics of nucleosomes over regulatory sequences and modifications of histone residues. The robustness of silencing is achieved through sub-optimization of many different factors such that in the presence of all these structures and processes a stable expression state is generated and maintained even though individual constituents are in constant flux.

## Data Availability

No datasets were generated or analysed during the current study.
